# Deep Transfer Learning with Enhanced Feature Fusion for Detection of Abnormalities in X-ray Images

**DOI:** 10.3390/cancers15154007

**Published:** 2023-08-07

**Authors:** Zaenab Alammar, Laith Alzubaidi, Jinglan Zhang, Yuefeng Li, Waail Lafta, Yuantong Gu

**Affiliations:** 1 School of Computer Science, Queensland University of Technology, Brisbane, QLD 4000, Australia; jinglan.zhang@qut.edu.au (J.Z.); y2.li@qut.edu.au (Y.L.); 2 Centre for Data Science, Queensland University of Technology, Brisbane, QLD 4000, Australia; 3 School of Mechanical, Medical and Process Engineering, Queensland University of Technology, Brisbane, QLD 4000, Australia; yuantong.gu@qut.edu.au; 4 ARC Industrial Transformation Training Centre-Joint Biomechanics, Queensland University of Technology, Brisbane, QLD 4000, Australia; 5 HMA Group, Brisbane, QLD 4172, Australia; wlafta@hmagroup.com.au

**Keywords:** musculoskeletal X-ray, deep learning, transfer learning, data scarcity, convolution neural network (CNN), machine learning, feature fusion, gradient-based class activation heat map

## Abstract

**Simple Summary:**

In this paper, we introduce a new technique for enhancing medical image diagnosis through transfer learning (TL). The approach addresses the issue of limited labelled images by pre-training deep learning models on similar medical images and then refining them with a small set of annotated medical images. Our method demonstrated excellent results in classifying the humerus and wrist, surpassing previous methods, and showing greater robustness in various experiments. Furthermore, we demonstrate the adaptability of the approach with a CT case, which showed improvements in the results.

**Abstract:**

Medical image classification poses significant challenges in real-world scenarios. One major obstacle is the scarcity of labelled training data, which hampers the performance of image-classification algorithms and generalisation. Gathering sufficient labelled data is often difficult and time-consuming in the medical domain, but deep learning (DL) has shown remarkable performance, although it typically requires a large amount of labelled data to achieve optimal results. Transfer learning (TL) has played a pivotal role in reducing the time, cost, and need for a large number of labelled images. This paper presents a novel TL approach that aims to overcome the limitations and disadvantages of TL that are characteristic of an ImageNet dataset, which belongs to a different domain. Our proposed TL approach involves training DL models on numerous medical images that are similar to the target dataset. These models were then fine-tuned using a small set of annotated medical images to leverage the knowledge gained from the pre-training phase. We specifically focused on medical X-ray imaging scenarios that involve the humerus and wrist from the musculoskeletal radiographs (MURA) dataset. Both of these tasks face significant challenges regarding accurate classification. The models trained with the proposed TL were used to extract features and were subsequently fused to train several machine learning (ML) classifiers. We combined these diverse features to represent various relevant characteristics in a comprehensive way. Through extensive evaluation, our proposed TL and feature-fusion approach using ML classifiers achieved remarkable results. For the classification of the humerus, we achieved an accuracy of 87.85%, an F1-score of 87.63%, and a Cohen’s Kappa coefficient of 75.69%. For wrist classification, our approach achieved an accuracy of 85.58%, an F1-score of 82.70%, and a Cohen’s Kappa coefficient of 70.46%. The results demonstrated that the models trained using our proposed TL approach outperformed those trained with ImageNet TL. We employed visualisation techniques to further validate these findings, including a gradient-based class activation heat map (Grad-CAM) and locally interpretable model-independent explanations (LIME). These visualisation tools provided additional evidence to support the superior accuracy of models trained with our proposed TL approach compared to those trained with ImageNet TL. Furthermore, our proposed TL approach exhibited greater robustness in various experiments compared to ImageNet TL. Importantly, the proposed TL approach and the feature-fusion technique are not limited to specific tasks. They can be applied to various medical image applications, thus extending their utility and potential impact. To demonstrate the concept of reusability, a computed tomography (CT) case was adopted. The results obtained from the proposed method showed improvements.

## 1. Introduction

X-ray medical images are widely recognised as powerful tools for identifying abnormalities in bone classification. They provide valuable information on the structure and condition of bones, helping to diagnose and treat various skeletal disorders. However, one of the most-significant challenges faced in the use of artificial intelligence (AI) algorithms for medical image analysis is the scarcity of data available for training and validation [1].

The limited availability of labelled medical images poses a substantial obstacle to the development of accurate and reliable AI models for bone classification tasks. Obtaining a large and diverse medical image dataset is crucial to the effective training of AI algorithms because the performance and generalisation capabilities of AI models can be compromised without sufficient data.

Addressing the issue of data scarcity in medical imaging is essential to unlocking the full potential of AI to improve bone classification accuracy and aid clinical decision-making. Researchers and practitioners continue to explore methods such as active learning (AL), synthetic data generation [2], generative data augmentation [3], transfer learning (TL) [4], and collaboration between institutions to overcome the challenges posed by limited medical image datasets. By expanding the availability and quality of annotated medical images, the performance and robustness of AI algorithms for bone classification will be enhanced and ultimately improve patient care in the medical imaging domain [5].

Despite advancements in medical applications and the progress made in computer vision, detecting abnormalities in the humerus and wrist using X-ray images remains a challenge [6]. The complexity of bone structures, subtle variations in abnormalities, and the inherent limitations of X-ray imaging techniques contribute to this challenge. Ongoing research and development efforts focus on using AI and machine learning (ML) techniques to improve the accuracy and efficiency of humerus and wrist abnormality detection in X-ray images. ML models can be trained on large datasets of annotated X-ray images, which enables them to recognise patterns and detect subtle pathological indicators.

By integrating AI and ML technologies into the field of orthopaedics, medical professionals can benefit from improved accuracy and efficiency when diagnosing and treating patients with bone conditions. Deep learning (DL) is a branch of AI techniques that has demonstrated exceptional abilities in accurately, reliably, and rapidly classifying medical images into binary and multiclass categories [7,8]. DL has become the gold standard in medical image analysis and has demonstrated remarkable performance in various areas, such as radiology [9,10], dermatology [11], pathology [12], and ophthalmology [13,14]. These applications, which span different medical fields, are based on human experience, thus making DL a valuable tool in a competitive domain.

The requirement for large amounts of labelled data is a significant challenge to the development of high-performing DL models. However, the scarcity and imbalance of medical image datasets pose significant challenges due to the cost effectiveness and time consumption associated with DL approaches. Despite these challenges, DL models have consistently demonstrated impressive performance in classifying medical images. Krizhevsky et al. [15] introduced a model based on a convolutional neural network (CNN) architecture that represented a significant milestone in the history of DL and computer vision. Their developing work demonstrated the potential of deep CNNs in image-classification tasks, thus setting new standards for accuracy and inspiring further research and innovation in the ImageNet Large Scale Visual Recognition Challenge (ILSVRC), a critical image classification competition. Several studies have successfully used TL techniques in DL models to address data scarcity. TL involves using models that were pre-trained on large-scale datasets and fine-tuning them on target medical image datasets [16]. This approach has been shown to be effective in improving the performance of DL models in various studies [1]. For example, Fang et al. [17] used fine-tuning and feature augmentation methods and an area under the curve (AUC) of 0.73. However, unbalanced data remain a limitation in certain studies [18]. Musculoskeletal radiographs (MURA) is a dataset designed specifically for musculoskeletal medical imaging, as it comprises a large collection of radiographs (X-rays). The dataset covers a diverse set of musculoskeletal abnormalities, as well as normal cases, which makes it a valuable resource for training and evaluating medical image classifiers [19]. The use of TL techniques for medical image classification has increased significantly, and this trend highlights the growing recognition of TL as a valuable strategy for handling data scarcity and improving the performance of DL models in the medical field.

CNNs have been widely used to classify input data as various states of disease [20]. CNNs’ deep architecture feedforward neural networks serve as the basis for many deep neural network models (DNN) in the medical field [20]. In addition to CNNs, other types of neural networks have also been used, such as recurrent neural networks (RNNs) with variations such as long short-term memory (LSTM), transformers, and generative adversarial networks (GANs) [21]. CNNs have proven to be particularly effective for image-processing and -classification tasks. One of their key strengths lies in their ability to extract meaningful patterns and characteristics from images regardless of scaling, mirroring, rotation, or translation [22]. This property makes CNNs highly suitable for medical image analysis, where the accurate identification and classification of image characteristics are crucial for diagnosis and treatment.

Furthermore, it should be noted that most studies that focus on humerus and wrist abnormalities do not thoroughly evaluate the “black box” explanation [23]. However, the lack of model explainability associated with black box methods is considered a significant obstacle to clinical adoption and user confidence [24]. To identify biases and ensure the reliability of DL applications, it is essential to explain the decision-making processes of the models. The use of TL is specifically recommended to address the issue of data scarcity and inconsistency in the medical field. TL leverages pre-trained DL models by using source datasets and fine-tuning them for target tasks. TL has had a positive impact on the medical field, especially in scenarios where limited data are available. Given the challenges of gathering medical imaging data, TL has become a crucial tool in medical image analysis. The ImageNet Large Scale Visual Recognition Challenge (ILSVRC-2012) competition dataset is widely recognised and widely used to improve the performance of various image-processing tasks, including classification, segmentation, and detection [25,26,27]. Although ImageNet has improved model performance, it is essential to note that medical images differ significantly from the natural images represented in ImageNet. These differences encompass various aspects, such as shape, colour, resolution, and dimensionality.

Models pre-trained on ImageNet are limited in terms of performance enhancement when dealing with medical images due to domain mismatch. Several authors have explained how TL using the same domain improved the performance of DL models in medical imaging applications [16,28,29].

Alternatively, the fusion technique could be used as an effective way to merge the features extracted by various CNN models for further enhancement. However, supporting the models’ results using the appropriate tools is necessary in order to trust the DL outcome [30].

The trade-off lies in the ability of DL models to leverage large amounts of data and learn complex patterns, whereas traditional techniques may have been more suitable for cases with limited data availability [31].

This paper aims to address the problem of data scarcity and the mismatch features of TL. Furthermore, it also addresses feature generalisation in training ML classifiers. Therefore, in this paper:

1. We propose a new TL approach to address the issue of data scarcity and the drawbacks of previous TL methods in medical imaging applications.
2. An improved feature fusion is proposed to increase trust in the final decision.
3. We employed two pre-trained ImageNet models for experimenting with two X-ray tasks to detect abnormalities in the humerus and wrist.
4. We applied a feature fusion strategy to train multiple ML classifiers in two different training scenarios.
5. We achieved an accuracy of 87.85%, an F1-score of 87.63%, and a Cohen’s Kappa coefficient of 75.69% in humerus classification. For wrist classification, our approach achieved an accuracy of 85.58%, an F1-score of 82.70%, and a Cohen’s Kappa coefficient of 70.46%.
6. We briefly review the most-recent DL techniques from the MURA dataset.
7. We explain how the decision was made to adopt two visualisation tools, i.e., gradient-weighted class activation mapping (Grad-CAM) and local printable model-agnostic explanations (LIME), to verify the robustness of the proposed method.
8. We show that the proposed TL approach and feature-fusion technique can be applied to various medical image applications, thus expanding their utility and potential impact, as demonstrated by adopting a computed tomography (CT) case that showed significant improvements in the results.

## 2. Related Work

This section provides an overview of the latest techniques used in the field. One such technique is the use of CNNs, which have demonstrated remarkable success in computer vision tasks and have become crucial for image classification. As mentioned above, the availability of large datasets and the time-consuming nature of training classifiers pose significant challenges to achieving optimal training results. Various techniques have been proposed to increase the size of the datasets, and one such strategy is active learning (AL). AL involves iteratively selecting the most-informative samples from an unlabelled dataset for annotation and model training. The primary goal of AL is to maximise the model’s performance while minimising the amount of labelled data required for training. This is particularly beneficial in scenarios where obtaining labelled data can be costly or time-consuming. The AL process relies on initially training the model on a small labelled dataset and then using a query strategy to determine which unlabelled samples should be selected for labelling. The query strategy is crucial, as it selects samples based on their potential impact on the model’s performance. There are several common query strategies used, including uncertainty sampling, query-by-committee, and information-density-based methods. After selecting and labelling these informative samples, the samples are incorporated into the training set to update the model using the newly labelled data. The training process for AL is often repeated over time until the model reaches a desired performance level or satisfies other specific criteria relevant to the desired application. This iterative approach helps the model learn from diverse and informative examples, which gradually improves its performance with fewer labelled samples. A key limitation of AL algorithms is that they are based on labelling one sample at a time. This means that, after each sample is labelled, the model needs to be retrained, which can be computationally expensive and time-consuming [32]. Researchers have thus been working on optimising this process to reduce the retraining burden.

For example, Wen et al. [33] conducted a study on using AL for nucleus segmentation in pathology images in which they investigated how AL performance improves for three different algorithm families: support vector machines (SVMs), random forest (RF), and CNNs. By employing AL, the researchers aimed to enhance the efficiency and accuracy of nucleus segmentation in medical imaging. Moreover, synthetic data generation is a powerful technique used to address data scarcity or privacy concerns by creating additional data artificially. In various domains, obtaining a sufficient amount of labelled data to train ML models can be challenging. Synthetic data generation offers a solution by generating new data points that are similar to the existing data, but are not direct replicas, thus expanding the dataset [3]. GANs have emerged as highly successful and versatile approaches that can find applications across various domains, including image generation, text generation, and video synthesis. GANs possess the remarkable ability to generate high-quality, diverse, and realistic synthetic data, making them invaluable for a wide range of tasks, including data augmentation, data synthesis, and creative applications [34]. Adar et al. [35] demonstrated the successful application of GANs for enhancing the classification performance for liver lesion classification by employing data augmentation techniques. In their study, conducted in 2018, the authors used GANs to increase the amount and diversity of training data, specifically for liver lesion classification. The authors observed a significant improvement in sensitivity and specificity compared to using traditional data-augmentation methods. Specifically, the classification performance increased from 78.6% sensitivity and 88.4% specificity (when using traditional enhancements) to 85.7% sensitivity and 92.4% specificity (when using GAN-generated data). This improvement can be attributed to the GAN’s ability to provide a more-diverse and -representative training dataset, allowing the classifier to better generalise and make more-accurate predictions on real-world liver lesion data.

Furthermore, Yi et al. [36] extensively explored and discussed the application of GAN image synthesis in various critical medical imaging domains. They highlighted the significant impact of GANs on improving medical image generation, analysis, and diagnostics across a range of applications. In the domain of brain magnetic resonance imaging (MRI), GANs have proven to be particularly valuable. Calimeri et al. [37] and Bermudez et al. [38] successfully used GAN-based image synthesis to generate realistic brain MRI scans. This synthetic data augmentation has led to the improved training of brain image analysis models and better performance in tasks such as segmentation and disease classification. For lung cancer diagnostics, Chuquicusma et al. [39] demonstrated the effectiveness of GANs in generating synthetic lung nodules and lesions. This data synthesis enabled the development and validation of robust and accurate lung-cancer-detection models, even when dealing with limited real-world data. High-resolution skin imaging is another domain where GANs have shown promise. Baur et al. [40] used GANs to synthesise high-resolution melanoma images. This approach enhanced the quality and diversity of the dataset used for training skin-cancer-detection models, leading to improved diagnostic accuracy and the early detection of skin cancer. While data augmentation is a powerful tool for improving model performance, its lack of interpretability can be a concern, especially in sensitive or critical applications. Ensuring transparency and explainability is essential for building trust and confidence in AI models, enabling users to comprehend why certain augmentations lead to improved performance. By incorporating human-understandable augmentation strategies and leveraging model interpretability techniques, researchers and practitioners can strike a balance between performance enhancement and interpretability, thereby making AI systems more trustworthy and responsible [41].

Additionally, Tahmina et al. [42] applied data augmentation in their study to detect humerus fractures. They used preprocessed images to increase the quality of the dataset, and the performance of their study was 78%.

Despite the promise of data augmentation, there are challenges that must be considered. Selecting appropriate augmentation techniques and parameters requires careful consideration. In addition, achieving the correct balance between augmenting and maintaining the integrity of the medical data is crucial to ensuring that the synthetic examples remain consistent with the real-world distribution of medical images. In this context, TL and pre-training are two alternative strategies for learning the low-level properties in CNNs. TL has proven to be an effective technique to train CNNs with limited data, thus improving the performance of DL models. TL enables one to leverage the knowledge and features learned from pre-existing models and apply them to new tasks or domains, thus reducing the need for extensive data collection and training time. By capitalising on pre-trained models, TL enables the efficient and effective training of CNNs even with smaller datasets. CNNs have been effectively trained using TL techniques in which the weights of pre-trained CNNs are used to classify other target images [20]. TL can be broadly categorised into two types: fine-tuned TL and feature extraction TL. The feature extraction TL approach employs a well-trained CNN model that was trained on a large dataset. The convolutional layers of the pre-trained model are frozen, whereas the fully connected layers are discarded. The frozen convolutional layers act as fixed feature extractors, which capture meaningful representations from the input images. These extracted features are then fed into a new classifier, which can be implemented using new fully connected layers or a supervised ML approach. During this type of TL, only the parameters of the new classifier are trained using the pre-learned features of the pre-trained CNN model [43].

This approach transfers knowledge from the pre-trained CNN model, which has learned rich representations from a large dataset, to the new medical task at hand. By using the extracted features and training only the classifier, feature-extracting TL enables efficient training with limited data, thus reducing the need for extensive computational resources and training time.

In contrast, the fine-tuning TL approach involves replacing the classifier layers while using the pre-trained CNN model that was trained on a large dataset as a base. In this approach, the convolutional and classifier layers are fine-tuned during the training process. The weights of the convolutional layers are initialised with the pre-trained weights from the CNN model, while the classifier layers start with random weights. The entire network is trained through this training process, allowing it to adapt and learn task-specific representations [44].

The fine-tuning TL approach is beneficial when the target task requires more-specialised knowledge and the available target dataset is more extensive. The model can learn task-specific features and improve its performance by updating the weights of the convolutional and classifier layers.

Both feature-extracting TL and fine-tuning TL have their advantages and are, thus, used based on the specific requirements of the task at hand. Feature-extracting TL is particularly useful when limited training data are available, as it leverages the pre-trained model’s learned features. Fine-tuning TL, however, can enhance performance by allowing the model to learn task-specific representations by updating both the convolutional and classifier layers. For this reason, this study used the fine-tuning TL type.

Various studies applied DL models to detect abnormalities in X-ray images, such as Ortiz et al. [45], who investigated three AI models to detect pneumonia in chest X-ray images; the authors used the feature-extraction technique with three different ML classifiers, and the accuracy of this study was 83.00% and with an 89% sensitivityfor radiomics, an accuracy of 89.9% with a 93.6% sensitivity for fractal dimension, and 91.3% accuracy with a 90.5% sensitivity for superpixel-based histon. Moreover, Canayaz et al. [46] implemented feature fusion by combining AlexNet and VGG19 models to classify COVID-19, pneumonia, and normal X-ray images; the authors’ approach achieved 99.38% accuracy. In addition, Rajinikanth et al. [47] used InceptionV3, which was pre-trained, to detect pneumonia in chest X-ray images. The authors used deep feature extraction and feature reduction with the Firefly Algorithm and multi-class classification using five-fold cross-validation; the results of the K-nearest neighbour (KNN) classifier demonstrated an accuracy of 85.18%. Furthermore, Rajinikanth et al. [48] also applied one-fold and two-fold training by using UNet lung section segmentation.

Indeed, DL models have been implemented in various studies to improve the detection of abnormalities in musculoskeletal images. Rajpurkar et al. [19] conducted a study using a dataset called MURA, consisting of 40,005 musculoskeletal images. Their research employed a DenseNet169 CNN architecture, as described in Huang et al. [49], whereby each layer was linked to all other layers in a feedforward fashion, thus achieving a deep network design. The model classified the images as abnormal if the prediction probability was greater than 0.5. The performance of the model was evaluated using two metrics: sensitivity and specificity. The sensitivity, which measures the ability of the model to identify true positives correctly, was 81.5%. The specificity, which measures the model’s ability to identify true negatives correctly, was 88.7%. These metrics indicate the model’s ability to detect normal and abnormal cases accurately. The model’s overall performance was also assessed using the area under the receiver operator characteristic (AUROC) metric, which considers the trade-off between sensitivity and specificity. The model achieved an AUROC of 92.9%, indicating its solid discriminative power. When diagnosing abnormalities in fingers and wrists, the model’s performance was roughly equivalent to that of the best radiologists. Despite the model’s agreement with the gold standard being similar to that of other radiologists, it was still relatively low. However, when diagnosing abnormalities in elbows, forearms, hands, humerus, and shoulders, the model’s performance was worse than that of the best radiologists [19].

To investigate this, Chada [50] conducted a study to evaluate the performance of three state-of-the-art CNN architectures, namely DenseNet169, DenseNet201, and InceptionResNetV2, on the MURA dataset. The researchers fine-tuned these CNN models using the Adam optimiser with a learning rate of 0.001. The evaluation was performed separately for humerus and finger images.

For the humerus task, the best performance was observed with the DenseNet201 model, which achieved a Cohen’s Kappa score of 76.4%. This indicated a substantial agreement between the predictions of the model and the ground-truth labels. For the images of fingers, however, the InceptionResNetV2 model demonstrated the best performance, obtaining a Cohen’s Kappa score (which assesses the agreement between the model’s predictions and the ground-truth beyond chance) of 55.5%. These results highlight the effectiveness of these CNN architectures in detecting abnormalities in musculoskeletal images, with performance variations depending on the specific anatomical areas. However, the performance of finger X-rays was less promising, undoubtedly due to the limitations of high inter-radiologist variation.

Another study focused on classifying proximal humerus fractures using the Neer classification system [51]. The researchers used a pre-trained ResNet-152 classifier that was fine-tuned for the specific task of classifying fractures. This approach leveraged the pre-trained weights of the ResNet-152 model and trained the classifier layers on the target dataset. Using this TL technique, the model accurately classified 86% of the proximal humerus fractures according to the Neer classification system. Despite the fact that the Neer classification is the most-regularly used technique for proximal humerus fracture classification, the reliability of this study needs to improve. The author in [51] assessed the diagnostic performance of CNNs with a cropped single-shoulder X-ray image, but this might not be applicable to the relative clinical scenario.

Furthermore, Lindsey et al. [52] investigated the detection of wrist fractures, comparing the performance of radiologists with and without the assistance of CNN models. The study aimed to assess how the use of CNN models affected radiologists’ diagnostic capabilities. The results indicated a marked increase in radiological performance when aided by CNN models, highlighting the potential of DL models as supportive tools in the field of fracture detection. However, the study had a number of drawbacks, such as the fact that the experiment was a review of the data performed through the web interface that simulated an image archiving and communication system (PACS) used by medical professionals for medical imaging. Furthermore, the accuracy of the physicians’ and the model’s diagnoses in this study was restricted to the determination of what is visible inside a radiograph. Finally, the diagnosed condition’s improvement or deterioration was influenced by factors other than DL accuracy in diagnosis.

Saif et al. [53] proposed a capsule-network-based approach to classify abnormalities in the musculoskeletal system. They conducted experiments by training their network on images of different sizes, specifically images of 64 × 64, 128 × 128, and 224 × 224 px. The goal was to determine the optimal image size to achieve an accurate classification, and the results indicated that, when using 224 × 224 px images, the network achieved the highest training accuracy (96%) for wrist radiography images. However, it is possible that the network’s performance in some studies was influenced by overfitting, a situation where the model becomes excessively tailored to the training data, which leads to the poor generalisation for unseen data.

However, in 2019, Varma et al. [54] introduced the MURA dataset, which included a private dataset of 93,455 lower-extremity radiographs with images of the foot, ankle, knee, and hip. This dataset was explicitly curated for abnormality detection using less-adequate extremity radiographs. To evaluate the performance of different CNN architectures, Varma et al. trained ResNet-50, DenseNet-161, and ResNet-101 on a subset of their private dataset. Despite the structural differences between these architectures, the authors found that performance did not vary significantly. However, the authors then proposed a comparison explicitly focused on the DenseNet-161 architecture, which was trained on both the ImageNet and MURA datasets, to assess the impact of TL on model performance. This comparison aimed to investigate the effect of TL, where a CNN model is initially pre-trained on a large-scale dataset (such as ImageNet) and then fine-tuned on a specific task or domain (in this case, the MURA dataset). Using pre-trained weights and pre-learned representations from the large-scale dataset, TL can potentially improve the performance of CNN models for the target task. However, the limitation of this study comes from the fact that it reviewed data with datasets from a single institution; thus, the performance of the authors’ models may differ in the real world when different images are used.

Furthermore, Kandel et al. [55] conducted a study using the MURA dataset to examine the performance of six CNN architectures, namely, VGG, Xception, ResNet, GoogLeNet, InceptionResNet, and DenseNet, to detect bone abnormalities. They compared models trained from scratch with pre-trained models using ImageNet and then fine-tuned them on the MURA dataset. The study’s results highlighted that TL has the potential to enhance model performance while reducing the susceptibility to overfitting. Among the five state-of-the-art CNNs evaluated for the MURA dataset, the humerus datasets achieved the highest precision (81.66%). Although the authors used the TL approach, the training-from-scratch approach’s poor performance could have been due to the number of images in the dataset, as well as the hyperparameter selection. The CNNs considered are distinguished by their incorporation of a significant number of trainable parameters (such as weights), and the number of images used to train these networks is insufficient to develop an effective model. Hyperparameters indicate the significance of the learning rate. Although the authors used a lower value of the learning rate in the fine-tuning technique to avoid significantly modifying the original weights of the designs, the training-from-scratch strategy may demand a higher value of the learning rate.

Feature fusion of DL techniques also was implemented by Bhan et al. [56] to classify fracture or non-fracture in the MURA dataset; the five pre-trained models were DenseNet-169, MobileNetV2 ResNet-50, ResNeXt-50 and VGG16, and then, these pre-trained models were combined in this study. The results of the feature-fusion approach were that the humerus achieved an 87.85% accuracy and a 75.72% Cohen’s Kappa, while the accuracy was 83.13% and a 66.25% Cohen’s Kappa for the shoulder. In the same study, the performance of the wrist classification was 86.65% accuracy and a 72.59% Cohen’s Kappa.

This literature review focused on the significant challenge caused by the limited availability of annotated data in the medical domain. The scarcity of annotated medical datasets prevents the full potential and effectiveness of DL algorithms. This challenge has motivated the main objective of this article, which was to explore strategies that can achieve greater performance with minimal data in the field of medical DL.

## 3. Materials and Methods

### 3.1. Dataset

The dataset used in this study is called MURA. It consists of X-ray images that represent seven different skeletal bones, namely the elbow, finger, forearm, hand, humerus, shoulder, and wrist. Each bone category is divided into two subclasses: positive (abnormal) and negative (standard). In total, the dataset contains 40,561 images. The dataset was partitioned into separate training and test sets, the details of which are presented in Table 1 below [19].

**Figure 1:**
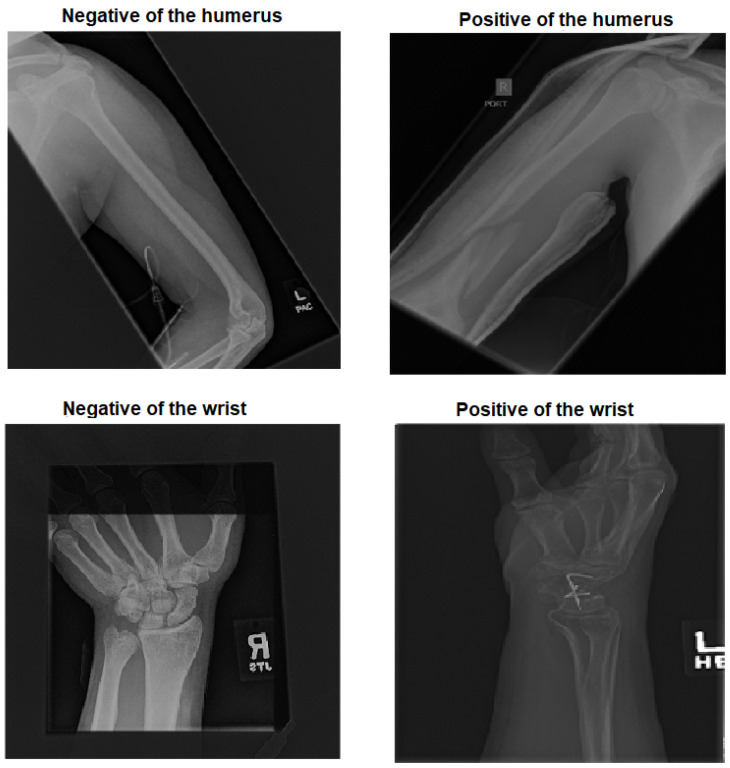
Four samples of the two tasks: the humerus and the wrist.

Two main categories were created from the dataset:

1. Target dataset: As shown in Figure 1, the categories of the humerus and wrist were specifically chosen as the target datasets for analysis and classification. These categories represent anatomical regions within the musculoskeletal system that are particularly interesting for medical image processing.
2. Source of TL: The source of TL is an important consideration in DL applications. In the context of TL, the source refers to the pre-trained models or datasets that are used as a starting point for training a new model on a target dataset. The rest of the MURA dataset was used as a source of TL, including the elbow, finger, forearm, hand and shoulder.

### 3.2. Proposed TL Technique

A large dataset was used in the TL stage to leverage the knowledge gained from this dataset and apply it to a smaller target dataset. One commonly used source for TL is pre-trained models that were trained on the ImageNet dataset. The ImageNet dataset comprises a vast collection of images categorised into 1000 classes, including various natural objects, people, plants, and animals. The pre-trained models derived from the ImageNet dataset have been widely used in multiple applications to address the challenge of limited data availability [57]. These models have demonstrated remarkable performance in object-detection and agriculture tasks, where the dataset encompasses diverse visual characteristics and requires robust feature-extraction capabilities. When the target task dataset shares relevant features with the ImageNet dataset, TL that uses pre-trained models becomes particularly valuable. However, it is essential to note that the ImageNet dataset consists primarily of colour images, which may not directly enhance the functionality of grayscale medical imaging. This distinction between colour and grayscale images highlights the need for careful consideration and customisation when applying TL techniques to medical imaging tasks, which often involve specific imaging modalities and grayscale representations [16].

**Figure 2:**
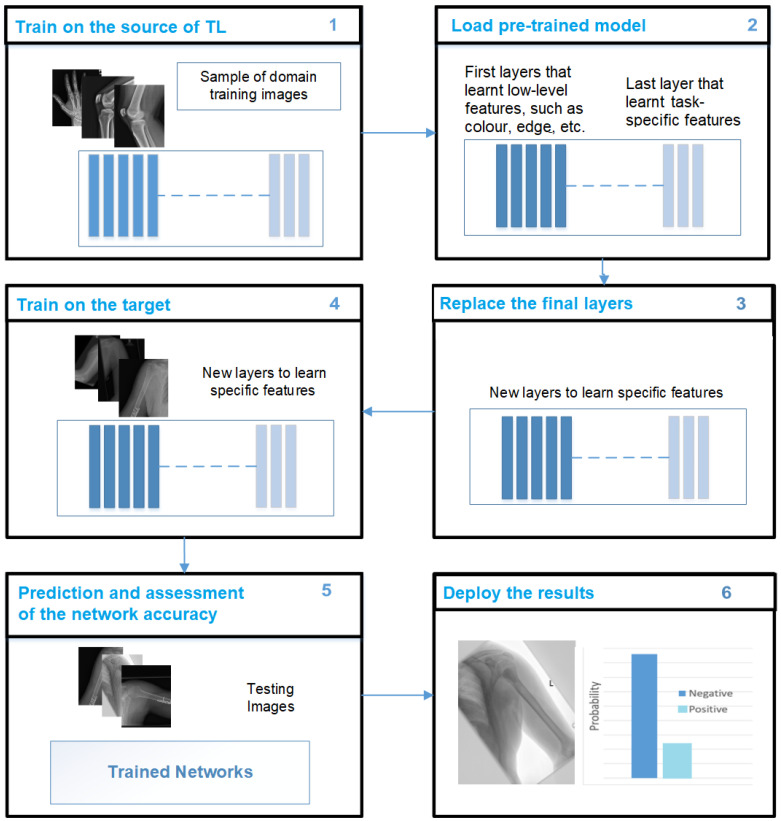
The proposed TL solution.

This work presents a new approach to TL and is called TL domain adaptation. This approach aims to address the challenge of limited annotated data and improve the performance of pre-trained ImageNet models in specific domains. The proposed method involves updating the features of the pre-trained models by incorporating in-domain images (source of TL) before fine-tuning the models on the target dataset (see Figure 2). This approach aims to leverage the knowledge and features learned from a wide range of musculoskeletal images to enhance the performance of the models. By incorporating various classes from the MURA dataset, the models can capture a greater understanding of musculoskeletal abnormalities and potentially generalise better to the target tasks of humerus and wrist abnormalities. One notable advantage of using the same image modality (X-ray) and having a common goal of detecting abnormalities across the MURA dataset and the humerus/wrist tasks is the similarities in the image characteristics and diagnostic objectives. These similarities enable a more-effective transfer of knowledge and features from TL source classes to the target humerus and wrist tasks. They also allow the models to capture relevant patterns, structures, and consistent abnormalities in different musculoskeletal areas. This approach improves the ability of the models to extract meaningful features and make predictions for abnormalities of the humerus and wrist, thereby improving performance and diagnostic precision. Figure 2 shows the workflow of the proposed method, which is as follows:

1. Step 1: Train the models on the source of TL (all MURA classes, except the target dataset).
2. Step 2: Load the pre-trained models.
3. Step 3: Replace the final layers (fully connected layer and classification layer).
4. Step 4: Train the model on the target (humerus or wrist) by freezing 70% of the layer of the model, and then, train the rest.
5. Step 5: Predict and assess the performance of the trained model in the target’s test images (humerus or wrist).
6. Step 6: Deploy the results.

This proposed TL eliminates the need for a large number of annotated images specific to the target task. This is beneficial when labelled data are scarce for the target task because valuable time and resources are saved. This study used two pre-trained models, both with and without the proposed TL approach. These models were chosen based on their strong performance on the ImageNet dataset, a benchmark for various computer vision tasks. The selection of diverse models allows for a comprehensive investigation of the effectiveness of the proposed TL technique. Table 2 presents the key characteristics of the selected models, including their sizes, depths, and image input sizes. By considering models with different architectures and specifications, this study aimed to assess the impact of TL in a variety of model configurations. This diversity allows a thorough evaluation of the effectiveness of the proposed TL technique and its potential application in various CNN models.

The limitation of this proposed TL is its need for a source of training, which requires time and computational resources. However, the ImageNet (S1) models have already been trained.

### 3.3. Training Scenarios

**Figure 3:**
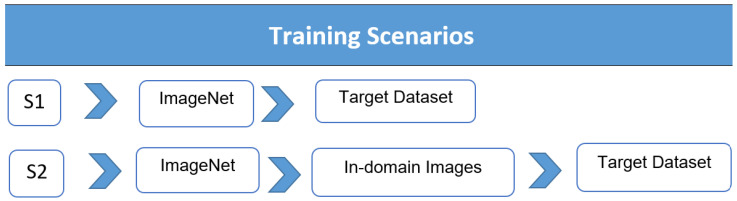
The two training scenarios.

Three groups were created from the dataset: training, validation, and testing. The training scenarios used in this study were calculated and conducted in the following ways (see Figure 3):

1. Scenario 1 (S1): TL from the ImageNet dataset was used to train the target dataset in the DL models.
2. Scenario 2 (S2): ImageNet (S1) models were trained with the TL source collection (in-domain images), and then, the models were trained on the target dataset. The training parameters included Adam optimisation, a minibatch size of 15, a maximum of 100 epochs, a shuffle for each epoch, and a starting learning rate of 0.001. An Intel (R) Core i7/32 GB/1 TB/Nvidia RTX A3000 12 GB were the GPU specifications used in the experiment. Matlab 2022a was used for the tests.

### 3.4. Deep Feature Fusion

The feature-fusion approach is used to improve overall performance by combining features from different DL models. It aims to capture and combine complementary information from multiple models to improve the representation of the features.

The first layers of each model learn basic features, such as colours, edges, and shapes, while the last layers learn all the features of the object. Therefore, we extracted the features from the last layers. Moreover, each DL model has its own structure and different filter sizes to learn the features, and combining them provides a better representation of the features. The two deep CNNs were trained and evaluated, and once trained, the models extracted the relevant features from the input data. These extracted features were then used to train the ML classifiers. In this process, the features extracted from both CNN models were combined into a single feature space. ML classifiers were trained to categorise and classify the abnormalities of the humerus or effectively classify the abnormalities of the humerus. The combination of the features of the two models allows for a more-comprehensive representation of the underlying patterns and characteristics present in the data.

The combination of the features of multiple models offers several advantages in ML classifiers. Combining the features extracted from various models makes a more-diverse and -comprehensive set of information available for classifiers to learn from. This approach allows ML classifiers to take advantage of the strengths and unique characteristics of each CNN model, resulting in a more-holistic understanding of the target tasks. The combination of trained models and the pooling of their features provides the final ML classifiers with the collective knowledge and discriminative power acquired from each model. This integration of features from multiple sources aims to improve the accuracy and robustness of the classification process. By considering a wider range of characteristics and capturing different aspects of the input data, classifiers can better capture the underlying patterns and nuances of abnormalities of the humerus and wrist.

**Figure 4:**
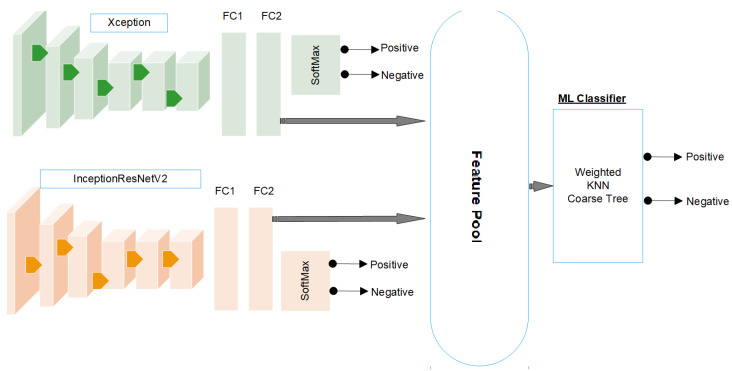
The feature-fusion process.

This study adopted various ML classifiers to use fused features. These classifiers included linear discriminant analysis, neural networks, coarse KNN, cubic SVM, the boosted tree, and the coarse tree. By applying multiple classifiers, each with its own strengths and characteristics, this study explored different approaches and identified the most-effective classifier for the given task, as shown in Figure 4.

### 3.5. Visualisation Techniques for Explainable Deep Learning Models

**Figure 5:**
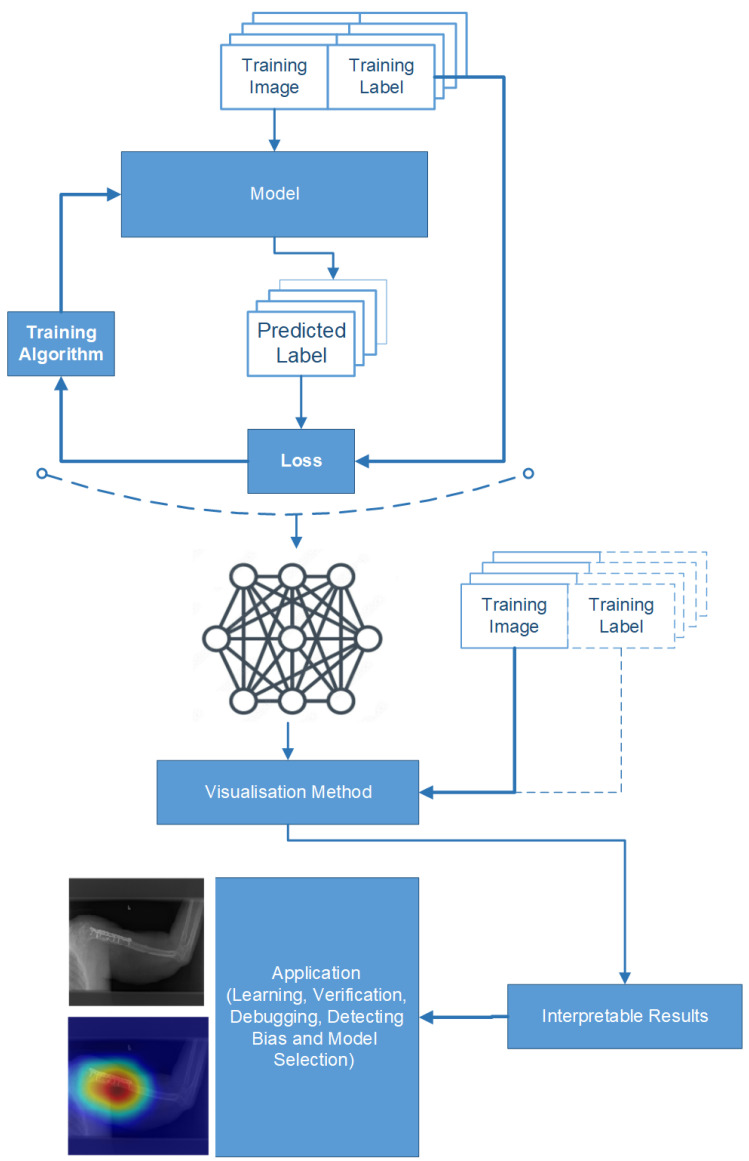
Workflow of visualisation techniques.

DL models are often referred to as “black boxes” due to the challenge of understanding why a model makes specific decisions. Gaining insight into their decision-making processes is essential to ensure confidence in DL models throughout the research and implementation stages. The methodologies used in this study have a wide range of applications, including model selection, debugging, learning, and bias assessment. One technique used to shed light on the predictions made by a network trained on image data is the use of test images, as depicted in Figure 5. These test images are used to clarify and understand the model predictions, and they also ensure that the models focus on the relevant regions of interest (ROIs) when making decisions. The gradient-weighted class activation mapping (Grad-CAM) visualisation technique, as well as local interpretable model-agnostic explanations (LIME) are interpretability techniques that explain the predictions of any ML model in an interpretable and understandable manner. Unlike Grad-CAM, however, which focuses on visualising important image regions, LIME can be applied to any input data type, including text and tabular data.

These techniques take advantage of the gradient by highlighting areas of the image that contribute significantly to the decision-making process of the model. The heat map is generated by computing the gradients of the target class score with respect to the feature maps in the final layer of the CNN.

Gradients involve taking partial derivatives of the loss function with respect to each parameter in the network. By iteratively computing and applying the gradients to update the parameters, the model learns to adjust its predictions and improve its performance on the given task. Gradients are then globally averaged together to obtain the importance weights for each feature map. A heat map is created by linearly combining the feature maps with their corresponding weights, which indicate the regions of the image that strongly influence the classification decision [58].

The visualisation techniques such as Grad-CAM and LIME aim to address the “black box” nature of DL models and enhance their interpretability and transparency. These techniques provide valuable information on the features of the image that the model considers crucial for decision-making. By analysing and understanding these critical regions, researchers can gain a deeper understanding of the model’s reasoning process and validate that the regions of influence align with the expectations. This helps to build confidence in the prediction of the model, especially in medical-image-analysis and diagnostic tasks.

## 4. Experimental Evaluation

This section focuses on the experimental assessment of the proposed TL method (S2) to detect any abnormalities in the humerus and wrist.

### 4.1. Evaluation Metrics

All models were evaluated based on various training scenarios, which used a comprehensive set of evaluation metrics, including accuracy, specificity, recall, precision, the F1-score, and Cohen’s Kappa. These metrics provide a comprehensive assessment of the model’s performance and ability to classify instances accurately. The evaluation metrics were calculated based on the values of the true negatives (TNs), true positives (TPs), false negatives (FNs), and false positives (FPs). The TN and TP values represent the correct classification of negative and positive examples, respectively, while the FP and FN values represent the incorrect classification of positive and negative examples, respectively.

These evaluation metrics collectively provide a comprehensive understanding of the performance of the model, thereby allowing an in-depth analysis of its strengths and weaknesses. By examining these metrics, researchers can assess the model’s capability to classify instances accurately and make informed decisions about its effectiveness in solving the target tasks. Each evaluation metric is presented as follows:

(1) $$\mathrm{Accuracy}=\frac{TP+TN}{TP+TN+FP+FN}$$

(2) $$\mathrm{Recall}=\frac{\sum \frac{TP}{TP+FP}}{num-classes}$$

(3) $$\mathrm{Specificity}=\frac{TN}{FP+TN}$$

(4) $$\mathrm{Precision}=\frac{TP}{TP+FP}$$

(5) $$F1-\mathrm{score}=2\times \frac{Precision*Recall}{Precision+Recall}$$

Cohen’s Kappa equation:

(6) $$\mathrm{Ko}=\frac{TP+TN}{TP+TN+FP+FN}$$

(7) $$\mathrm{Kpositive}=\frac{\left(TP+FP)(TP+FN\right)}{{\left(TP+TN+FP+FN\right)}^{2}}$$

(8) $$\mathrm{Knegative}=\frac{\left(FN+TN)(FP+TN\right)}{{\left(TP+TN+FP+FN\right)}^{2}}$$

(9) Ke=Kpositive+Knegative

Cohen’s Kappa score=

(10) $$\frac{\mathrm{Ko}-\mathrm{Ke}}{1-\mathrm{Ke}}$$

### 4.2. Experimental Evaluation of End-to-End DL Models

Two DL models were tested with the help of two training scenarios, as shown in Table 3 and Table 4.

**Figure 6:**
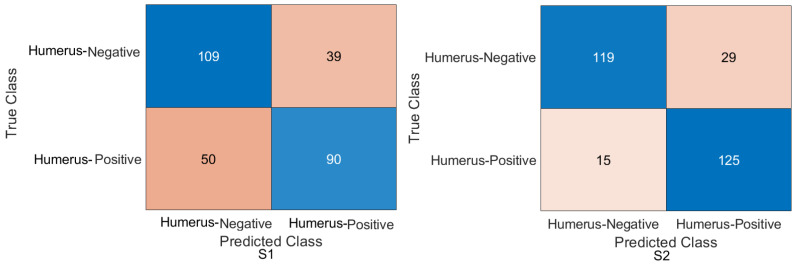
Confusion matrix of the Xception model on the test set with two different training scenarios of the humerus task.

**Figure 7:**
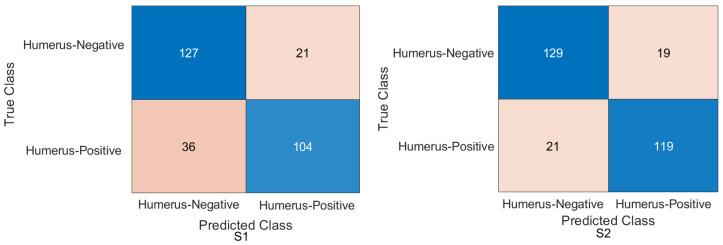
Confusion matrix of the InceptionResNetV2 model on the test set with two different training scenarios of the humerus task.
  The Grad-CAM visualisation technique was used to explain the black box nature of the DL models for the two training scenarios. In this section, we used trained models with the test images to calculate the confidence value for Grad-CAM, and two examples are presented to illustrate the performance of the models. The first example is shown in Figure 8, which includes three situations with positive samples. The heat maps reveal the behaviour of the S1 and S2 models when identifying the test samples and focussing on the region of interest (ROI). For S1, the misusing model, the heat map indicates that the model identified the test sample, but concentrated on areas outside the ROI. This suggests that S1 may not accurately capture the essential features within the ROI. However, the proposed TL (S2) method accurately identified the sample with a high confidence value, and the associated heat map focuses on the ROI. This demonstrates the effectiveness of S2 in capturing the relevant information within the ROI. The second example shown in Figure 9 presents negative samples and exhibits the same comparison of S1 and S2. In this case, S2 successfully identifies the samples with a high confidence value, and the heat map targets the ROI. Although it has a low confidence value, S2 still correctly identifies the samples, indicating its robustness. In contrast, the heat map produced by S1 reveals that it focuses on regions outside the ROI, which suggests that it is unable to capture important features within the intended area. These two examples highlight how the proposed TL (S2) method significantly improves the results compared to the misusing model. Although S1 may demonstrate accurate predictions, its low confidence level and lack of an ROI-focused approach make it unreliable. On the contrary, S2 achieves accurate predictions with high confidence values and effectively focuses on the ROI, thus showcasing the enhancement provided by the proposed method (S2).

1. Humerus task: As shown in Figure 6 and Figure 7, the confusion matrix was initially calculated for all training cases. The assessment metrics were derived from the confusion matrix values, which provided a detailed breakdown of the model classifications. The data demonstrated the performance of the different scenarios, with S2 achieving the best overall results. S2 obtained an accuracy of 84.72%, and the recall, also known as the true positive rate, was 89.29%. The precision, which measures the accuracy of positive predictions, was 81.17%, while the specificity, which represents the true negative rate, was 80.41%. The F1-score, which balances precision and recall, was 85.03%, providing an overall measure of the performance of the model, and Cohen’s Kappa, which assesses the agreement between the model predictions and the ground-truth beyond chance, was 69.83% with the Xception DL model. S2 with the InceptionresNetV2 model had an accuracy of 86.11%, a recall of 85.00%, a precision of 86.23%, a specificity of 87.16%, an F1-score of 85.61%, and a Cohen’s Kappa of 72.20%. In comparison, S1 with the Xception model achieved a precision of 69.10%, indicating a lower general accuracy of the predictions. The recall, precision, specificity, and F1-score for S1 were 64.29%, 69.77%, 73.65%, and 66.91%, respectively. Cohen’s Kappa for S1 was 38.02%. In contrast, S1 with the InceptioiresNetV2 model achieved an accuracy of 80.21%. Meanwhile, the recall, precision, specificity, F1-score, and Cohen’s Kappa for S1 were 74.29%, 83.81%, 85.81%, 78.49%, and 60.27%, respectively.

**Figure 8:**
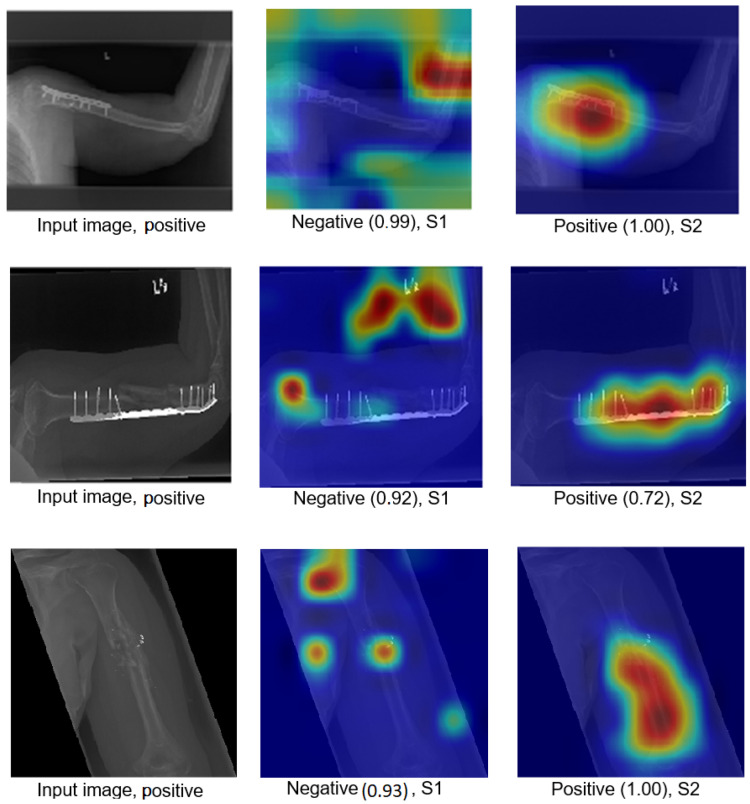
Grad-CAM and score Grad-CAM for the humerus X-ray images. The correct classification is positive.

**Figure 9:**
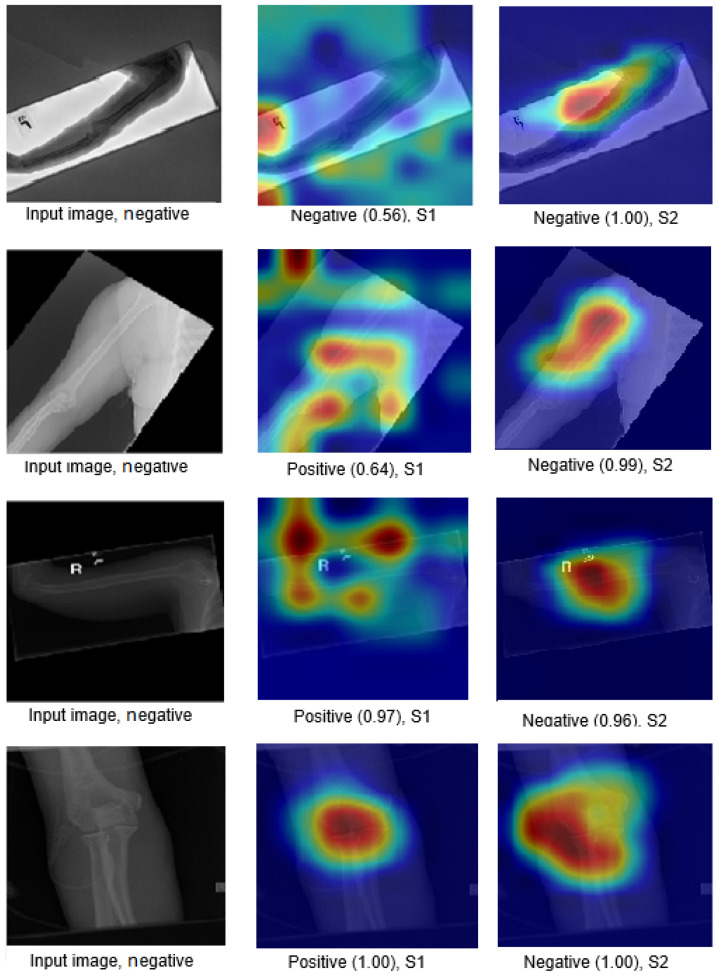
Grad-CAM and score Grad-CAM for the humerus X-ray images. The correct classification is negative.
2. Wrist task: The confusion matrix was calculated for all training cases in both models of DL, and the results are shown in Figure 10 and Figure 11. Using the values of the confusion matrix, various assessment measures were derived. The results demonstrate that S2 outperforms the other model with the Xception DL model, and S2 achieves the highest performance. Specifically, S2 achieves an accuracy of 84.07%, a recall of 73.56%, a precision of 88.93%, a specificity of 92.58%, an F1-score of 80.52%, and a Cohen’s Kappa of 68.11%. In contrast, S1 achieves an accuracy of 69.10%, a recall of 64.29%, a precision of 69.77%, a specificity of 73.65%, an F1-score of 66.91%, and a Cohen’s Kappa of 38.02%.
  As in the InceptionresNetV2 DL model, the results demonstrate that S2 achieves an accuracy of 82.85%, a recall of 67.80%, a precision of 91.74%, a specificity of 95.05%, an F1-score of 77.97%, and a Cohen’s Kappa of 64.45%. S1 achieves an accuracy, recall, and precision of 80.21%, 74.29%, and 83.81%, respectively. Meanwhile, the different performance metrics obtain a specificity of 85.81%, an F1-score of 78.49%, and a Cohen’s Kappa of 60.27%.
  These results highlight the superior performance of S2 compared to S1. S2 shows superior precision, recall, specificity, and F1-score, highlighting its classification ability. The higher Cohen’s Kappa value suggests better agreement between the predicted and true labels. On the contrary, S1 exhibits lower performance in all assessment measures, indicating its limitations in accurately classifying data.

**Figure 10:**
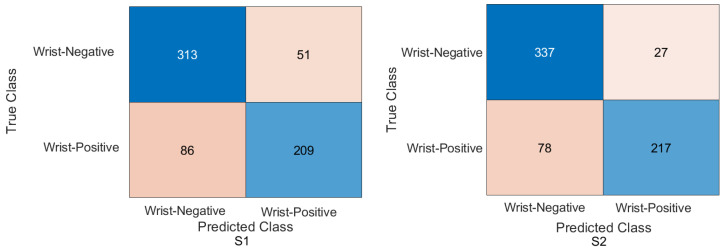
Confusion matrix of the Xception model on the test set with two different training scenarios of the wrist task.

**Figure 11:**
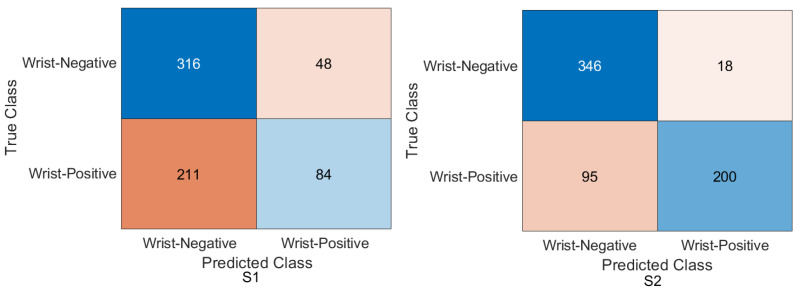
Confusion matrix of the InceptionResNetV2 model on the test set with two different training scenarios of the wrist task.
  Figure 12 illustrates how S2 confirms the proposed method (S2) for the wrist task, as well as with Grade-CAM.
  In terms of LIME and score LIME, Figure 13 provides a comparison between S1 and S2. For S1, the figure illustrates that the model mispredicts the test sample, where the high-intensity area is outside the ROI. This misprediction is evident from the LIME visualisation, highlighting the incorrect area as having maximum intensity. The confidence level of the model in this prediction is not specified. In contrast, S2 shows a significant improvement in accuracy. The model predicts the input sample confidently with a confidence level of 100%. The LIME visualisation confirms that the model correctly identifies the ROI, as the maximum intensity value is assigned to the relevant area. This example serves as a demonstration of the effectiveness of the proposed TL (S2) method. By incorporating the proposed approach, the model prediction is transformed from an incorrect prediction (as observed in S1) to an accurate prediction (as demonstrated in S2). The visualisation provided by LIME further supports this improvement by highlighting the crucial regions that contribute to the correct prediction. In general, the comparison of S1 and S2 using LIME and score LIME emphasises the efficacy of the proposed TL (S2) method in improving the accuracy and reliability of the prediction of the model, particularly by ensuring that the ROI is correctly identified and considered during the decision-making process.

### 4.3. Experimental Evaluation of Deep Feature Fusion for the Humerus Task

Several ML classifiers were trained using features extracted from two models, such as the decision tree, linear discriminant analysis, naive Bayes, support vector machine (SVM), coarse KNN, K-nearest neighbour, logistic regression, and neural networks.

**Figure 14:**
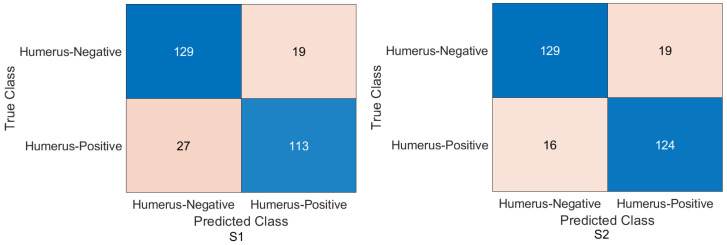
Confusion matrix of the two models and the coarse KNN classifier on the test set with two different training scenarios of the humerus task.

Interestingly, the coarse KNN classifier exhibited excellent performance in both scenarios. Figure 14 illustrates the confusion matrix using the cotoNN classifier for each scenario. However, the results presented in Table 5 show that both S1 and S2 significantly improved the results compared to the baseline. Specifically, S2 achieved an accuracy rate of 87.85%, a recall of 88.57%, a precision of 86.71%, a specificity of 87.16%, an F1-score of 87.63%, and a Cohen’s Kappa of 75.69%. These metrics indicate a high level of performance and reliability for S2. On the contrary, S1, which was trained using the same coarse KNN classifier, achieved an accuracy of 84.03%, a recall of 80.71%, a precision of 85.61%, a specificity of 87.16%, an F1-score of 83.09%, and a Cohen’s Kappa of 67.98%. Although slightly lower than S2, these results nevertheless demonstrated the effectiveness of S1 in improving overall performance compared to the baseline. The comparison between S1 and S2 using the coarse KNN classifier emphasised the superior performance of the proposed method (S2). Both scenarios significantly improved the accuracy, recall, precision, specificity, F1-score and Cohen’s Kappa. These results highlighted the effectiveness of the proposed TL (S2) method in enhancing the overall performance of classifiers.

**Figure 15:**
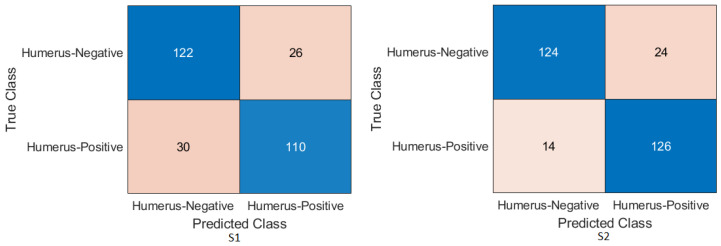
Confusion matrix of the two models and Gaussian naive Bayes classifier on the test set with two different training scenarios of the humerus task.

In comparison with another ML classifier, Table 6 displays the result for the humerus task with the Gaussian naive Bayes ML classifier, and Figure 15 confirms the result with the confusion matrix.

### 4.4. Experimental Evaluation of Deep Feature Fusion for Wrist Task

**Figure 16:**
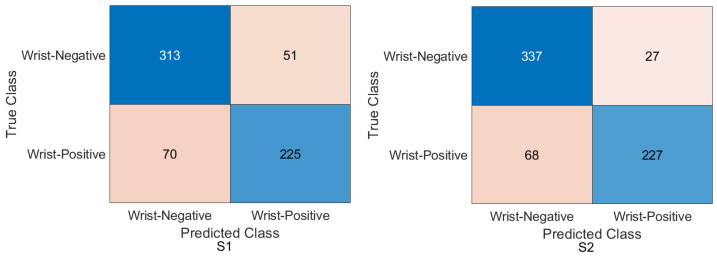
Confusion matrix of the two models and coarse KNN classifier on the test set with two different training scenarios of the wrist task.

The features extracted from the two models for the wrist task were used to train various ML classifiers, including the decision trees, linear discriminants, naive Bayes, support vector machine (SVM), coarse KNN, K-nearest neighbour, logistic regression, and neural networks. The features were obtained from the two training scenarios, and the outcomes of the classifiers showed a similar pattern. In particular, the coarse KNN classifier exhibited exceptional performance in both scenarios. The confusion matrix was initially constructed using the coarse KNN classifier for each situation, as shown in Figure 16. However, the results presented in Table 7 reveal that both S1 and S2 significantly outperformed the baseline in terms of improving the overall results.

Specifically, S2 achieved an accuracy of 85.58%, a recall of 76.95%, a precision of 89.37%, a specificity of 92.58%, an F1-score of 82.70%, and a Cohen’s Kappa of 70.46%. These metrics indicated high precision and performance for S2 on the wrist task. In comparison, S1, which was trained using the same coarse KNN classifier, achieved an accuracy of 81.64%, a recall of 76.27%, a precision of 81.52%, a specificity of 85.99%, an F1-score of 78.81%, and a Cohen’s Kappa of 62.64%. Although slightly lower than S2, these results demonstrated the effectiveness of S1 in improving the overall performance compared to the baseline.

The comparison between S1 and S2 using the coarse KNN classifier emphasised the superior performance of the proposed TL (S2) method. Both scenarios significantly improved the accuracy, recall, precision, specificity, F1-score, and Cohen’s Kappa. These results highlighted the effectiveness of the proposed approach in improving the overall performance of ML classifiers in the wrist task.

**Figure 17:**
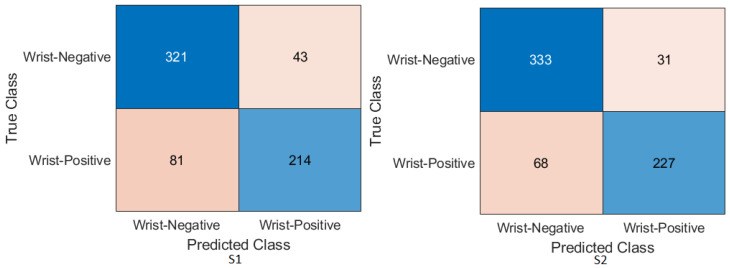
Confusion matrix of the two models and the linearSVM classifier on the test set with two different training scenarios of the wrist task.

Table 8 shows the result for the feature fusion for the wrist task with the linearSVM ML classifier on the test MURA dataset for the wrist task, and Figure 17 confirms the result with the confusion matrix.

Table 5 and Table 7 provide some key conclusions:

1. The results obtained from both the humerus and wrist tasks demonstrated that, in Scenario 1 (S1), the performance was inferior compared to Scenario 2 (S2), despite the employment of feature-fusion techniques in both cases. However, it is worth noting that the application of feature fusion without addressing the underlying problem of data scarcity might have weakened the fusion process due to inadequate feature representation.
2. Significantly, it should be noted that, in Scenario 2 (S2), the fusion process exhibited remarkable improvement once the data scarcity problem was addressed. This enhancement can be attributed to the use of the high-quality features extracted by the models. The introduction of sufficient and diverse data allowed for a more-robust and -comprehensive representation of the underlying information, resulting in improved fusion performance.
3. By integrating features from different models or sources, feature fusion plays a crucial role in diversifying the representation of a model, thus reducing the risk of overfitting. This process involves incorporating diverse information, which allows the model to learn from multiple perspectives and reduces its dependence on specific features or patterns in the training data. Consequently, the model becomes more flexible and capable of effectively generalising its knowledge to unseen data instances. Moreover, feature fusion contributes significantly to the achievement of high generalisation performance. Generalisation refers to the model’s ability to perform well on data samples that lie beyond the training set.

### 4.5. Comparison Against the State-of-the-Art Methods

Our proposed method (S2) obtained good results compared to many studies, as shown in Table 9 and Table 10.

### 4.6. Robustness of Our Proposal

**Figure 18:**
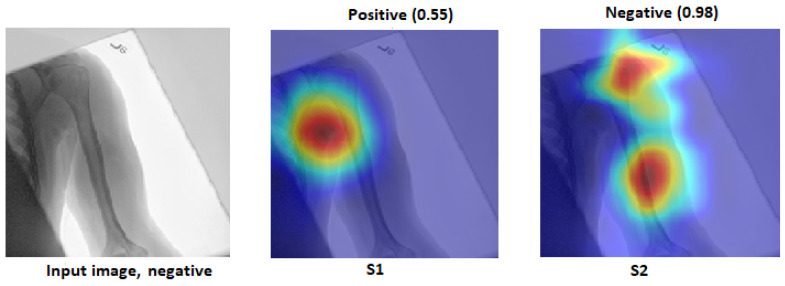
Grad-CAM and score Grad-CAM for the humerus X-ray images. The correct classification is negative.

This section demonstrates the robustness of our methodology in the following ways:

1. Improvement of results:
  Figure 18 and Figure 19 demonstrate the comparison between S1 and S2 and visually depict the significant contrast in the prediction outcomes, highlighting the remarkable improvement achieved by S2 over S1. S2 successfully transformed incorrect predictions into correct predictions and did so with a high confidence value.
  The two figures provide concrete evidence of how the proposed TL (S2) method substantially improved the performance of the model by accurately identifying the ROI. In the visualisations, it is evident that S2 precisely identified the crucial areas within the image that influenced the correct prediction. This focus on the ROI was instrumental in achieving the improved accuracy and reliability in the model predictions. The comparison between S1 and S2 is compelling proof of the effectiveness of the proposed TL (S2) method. It demonstrated the significant impact that the consideration of the ROI and the implementation of appropriate techniques can have on enhancing prediction outcomes. The improved performance of S2, along with the high confidence value associated with the correct predictions, highlighted the success of the proposed TL (S2) method in improving both the accuracy and the reliability of the model.
  In particular, Grad-CAM and score Grad-CAM in Figure 18 display a negative case of the humerus as an input image. It is obvious in the S1 of ImageNet that the model incorrectly classified. However, the TL approach (S2) correctly classified with a confidence of 98.00%. On the other hand, Figure 19 displays how our approach TL (S2) correctly classified the input images in both cases (humerus and wrist), while ImageNet (S1) misclassified them.

**Figure 19:**
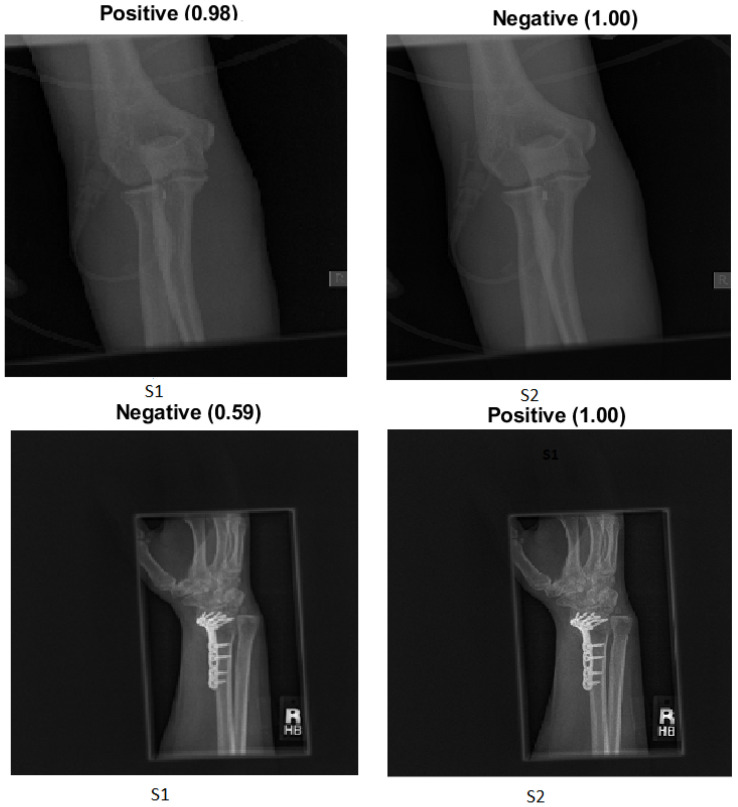
Comparison of S1 and S2, where S1 misclassified the samples and S2 correctly classified them.
2. Changes to sensitivity:
  To demonstrate the robustness of our technique (S2), S2 was tested against various alterations. Figure 20 and Figure 21 show how a small adjustment, such as removing the printed letters from the red circle, can affect the performance of S1. The estimate was made outside of the ROI and went from being accurate to erroneous before and after being adjusted. However, S2 demonstrated consistent performance by correctly predicting the samples with a high prediction and correctly identifying the ROI.
  Figure 20 and Figure 21 illustrate that, despite removing the letter from both tasks (humerus and wrist), TL (S2) was not affected by these changes. However, ImageNet (S1) was affected by these changes and changed the classification from positive (0.86) to negative (0.99) in the humerus task when the input image was positive and from negative (0.75) to positive (0.89) in the wrist task, despite the input image being negative.

**Figure 20:**
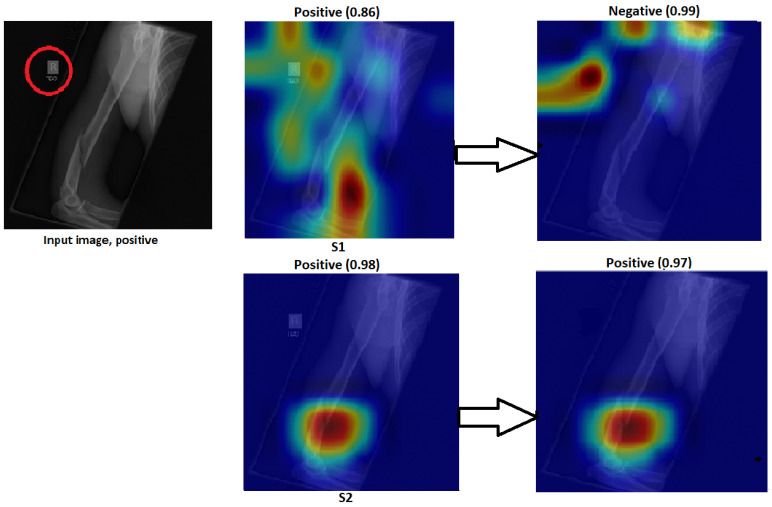
The effect of certain modifications made by eliminating the letters in the red circle. Positive is the correct classification in the humerus task.

**Figure 21:**
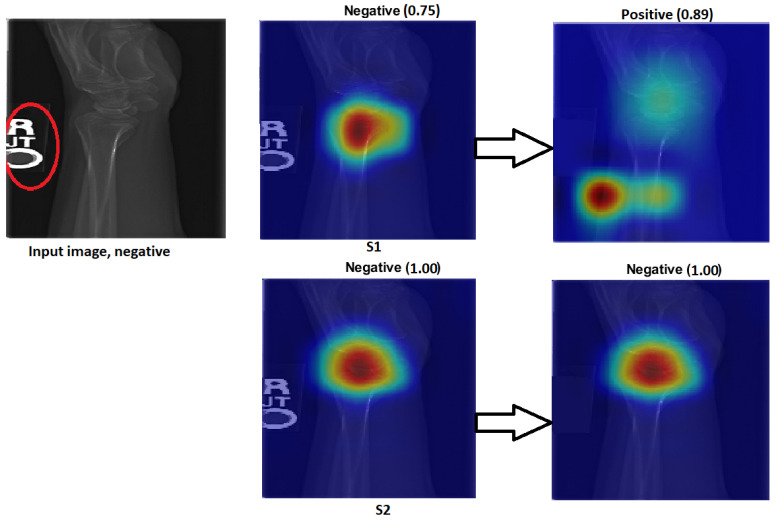
The effect of certain modifications made by eliminating the letters in the red circle. Negative is the correct classification in the wrist task.
3. Assessment of confidence:
  - S1 had a high score with high confidence and correctly recognised the sample in Figure 22 despite not being focused on the ROI, which is interesting. Although the confidence level was high, S1 cannot be relied upon because the Grad-CAM visualisation suggested the opposite to be true. The sample was incorrectly classified with a high confidence value by eliminating the background.
  To approve our approach of TL (S2) by removing the background in Figure 22, TL (S2) still had more confidence with 100% accuracy with the background and without rather than ImageNet (S1), which failed when the background was removed and directly changed from (0.87) positive to (0.77) negative with the positive input image.

**Figure 22:**
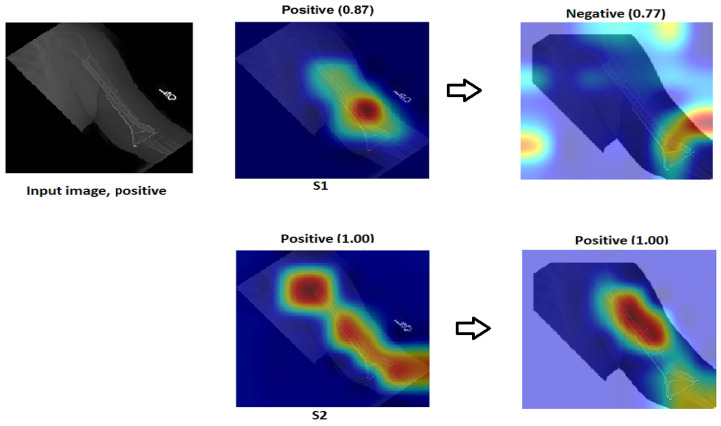
Comparison of S1 and S2. The right classification is positive.
  - Low score: Several test samples are shown in Figure 23, where S1 and S2 successfully identified them. S1 anticipated the samples with low confidence values, but these samples were unreliable because the model did not guarantee them, particularly the samples with confidence values in the 0.50 range. However, S2 displayed a high confidence score that corresponded to the confidence expectation.
  In Figure 23, we can see the obvious difference in the score of classification between ImageNet (S1) and TL (S2); TL (S2) correctly classified with 100% confidence. Meanwhile, ImageNet (S1) correctly classified some images with a low score of confidence.
4. Better feature representation:
  -Fusing two or three DL models enhances the feature representation for ML classifiers and improves the overall performance. Figure 24 shows that one model missed the classification and made incorrect feature selections, while the other model correctly classified the sample. Employing the feature-fusion technique can significantly reduce the chances of misclassification.
  Figure 24 confirms the feature-fusion technique, with the positive humerus and positive wrist that InceptionResNetV2 correctly classified in the TL (S2). However, the same model (InceptionResNetV2) incorrectly classified them (humerus and wrist) with ImageNet (S1).

**Figure 23:**
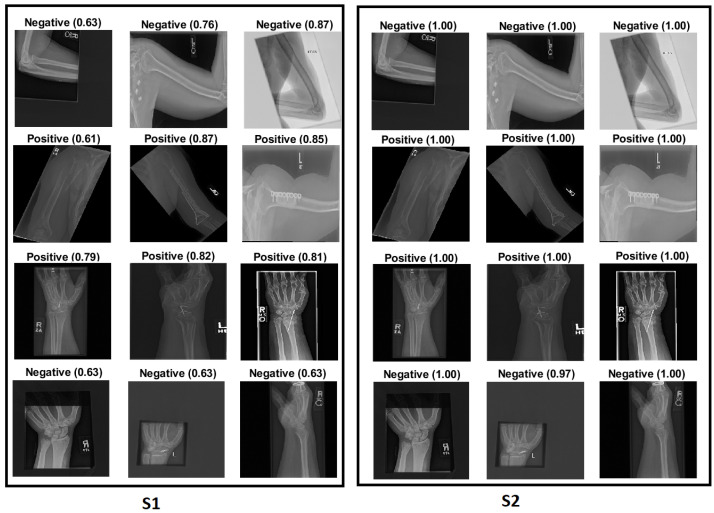
Comparison of S1 and S2, where S1 and S2 correctly predicted the samples, but had varying confidence scores.

**Figure 24:**
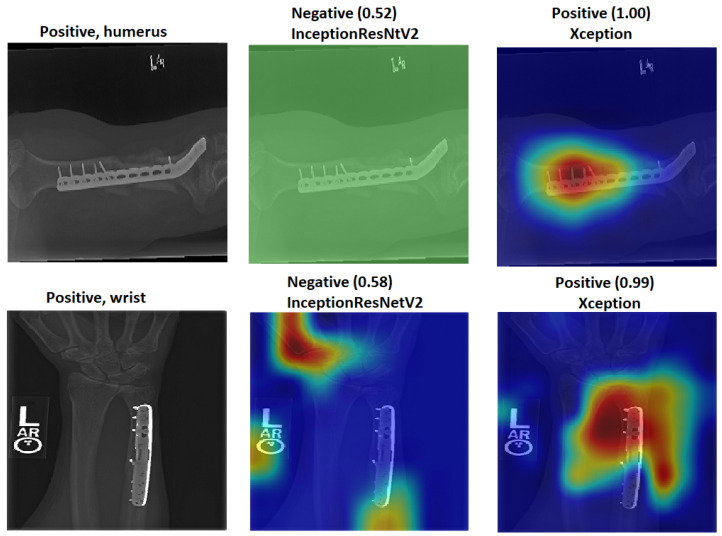
Comparison of InceptionResNetV2 and Xception. The correct classification is positive.

## 5. Reusability of the Proposed Solution

**Figure 25:**
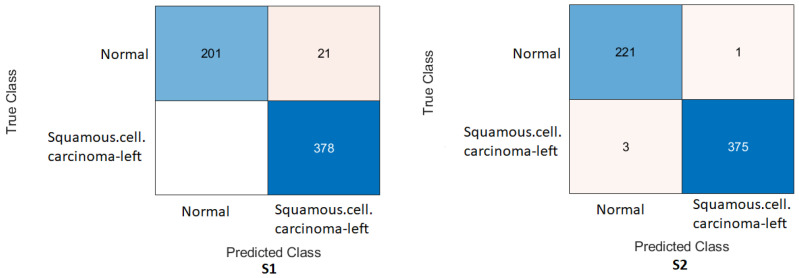
Confusion matrix of the Xception model on the test set with two different chests CT scan task training scenarios.

**Figure 26:**
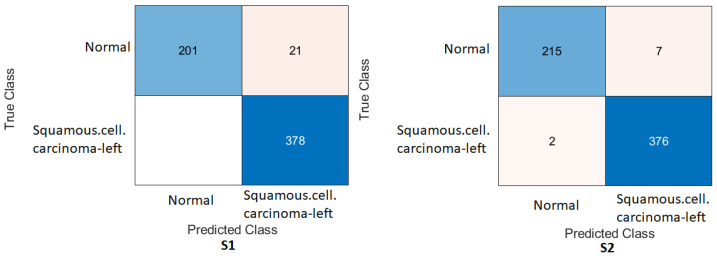
Confusion matrix of the InceptionResNetV2 model on the test set with two different chest CT scan task training scenarios.

To approve our TL (S2) approach, we applied our proposed method (S2) to another dataset (chest CT scan), which includes two classes (normal and squamous.cell.carcinoma-left). First, we trained the dataset with Xception and InceptionResNetV2 from scratch to generate S1. Next, we used our source of X-rays without any images of new data (chest CT scan). Table 11 demonstrates the performance for both scenarios in the two models. Figure 25 and Figure 26 display the confusion matrix for both DL models (Xception and InceptionResNetV2) on a test of the chest CT scan dataset.

Figure 25 displays that the Xception model with ImageNet (S1) misclassified (21) images of a normal class, when with the TL (S2), only (1) of the normal class and (3) of the squamous.cell.carcinoma-left were misclassified. Meanwhile, Figure 26 clarifies that the InceptionResNetV2 model misclassified (21) images of the normal class in the ImageNet (S1), and our approach with TL (S2) misclassified (7) images of the normal class and (2) images of the squamous.cell.carcinoma-left.

**Figure 27:**
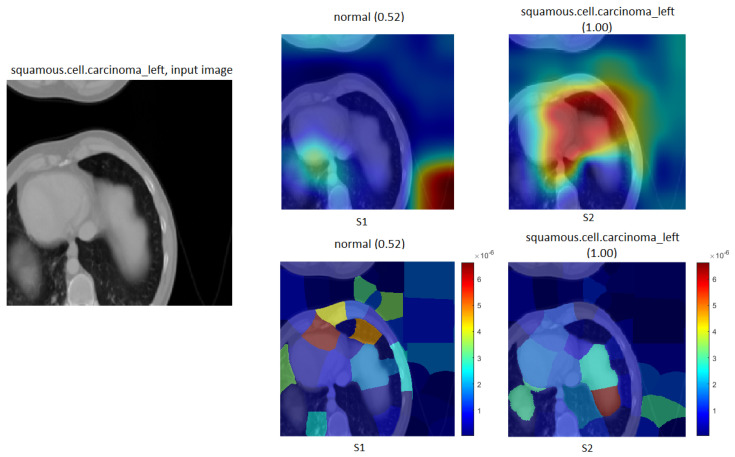
Grad-CAM in the first row and LIME in the second row for chest CT scan images. The correct classification is squamous.cell.carcinoma-left.

Furthermore, the Grad-CAM and LIME tools were applied to the CT scan dataset to confirm our proposed method of TL (S2), as shown in Figure 27.

Specifically, the model in Figure 27 demonstrates that ImageNet (S1) incorrectly classified the squamous.cell.carcinoma-left as the input image. Meanwhile, our approach of TL (S2) confirmed the squamous.cell.carcinoma-left images with a confidence of 100% using the Grad-CAM and LIME tools of the visualisation technique.

## 6. Conclusions and Future Work

In this paper, a robust technique was introduced for identifying abnormalities in X-ray images of the humerus and wrist. The technique addresses the challenge of domain mismatch between coloured natural images and grayscale X-ray images by training two pre-trained models from ImageNet (S1) on in-domain X-ray images specifically related to the elbow, finger, forearm, hand, humerus, and wrist. These models were then fine-tuned using a dataset specific to the tasks of the humerus and wrist.

The proposed method (S2) was compared with two other training conditions. The first condition (S1) involved using ImageNet (S1) directly on the intended dataset without addressing the domain mismatch. The second condition consisted of training multiple ML classifiers using the fused features extracted from the two models obtained in each scenario.

By overcoming the domain mismatch and training the models on in-domain X-ray images, the proposed method (S2) aimed to improve the accuracy and effectiveness of anomaly detection in humerus and wrist X-ray images. A comparison against other training conditions provided information on the benefits of the proposed approach (S2) in capturing relevant features and improving anomaly detection performance. From this research, we concluded the following:

1. The test dataset consisted of pure MURA images without any preprocessing applied. It is worth noting that many state-of-the-art studies use various preprocessing techniques to enhance image quality and improve their results. However, our approach outperformed these state-of-the-art methods despite not employing preprocessing techniques on the test dataset. By demonstrating superior performance without preprocessing, our approach highlights the effectiveness of the proposed methodology (S2) in accurately detecting anomalies in MURA images. It suggests that our models’ robust feature extraction and classification capabilities can capture the relevant information directly from the raw MURA dataset.
2. The results obtained from S2 demonstrate the effectiveness of the proposed TL approach (S2) in reducing the mismatch between the two domains. By training the models on in-domain X-ray images and then fine-tuning them on the humerus and wrist tasks datasets, the TL method effectively bridged the gap between the coloured natural images and the grayscale X-ray images. The reduced mismatch in the domain is reflected in the improved performance of S2 compared to S1 and other training conditions. The models trained using S2 exhibited enhanced prediction accuracy and demonstrated the ability to correctly identify the ROIs in the X-ray images. This reduction in domain mismatch can be attributed to the transfer of knowledge and features from the pre-trained ImageNet (S1) models to the specific tasks of humerus and wrist anomaly detection.
3. For some models of ImageNet (S1), despite specific models showing comparable or higher performance in the evaluation metrics, the visual confirmations provided by Grad-CAM and LIME emphasised the superiority of S2 in accurately detecting and focusing on the relevant ROI. These visualisation techniques added a layer of confidence to the outcomes obtained by S2.
4. The proposed approach (S2) is not limited to the specific dataset used in this study; it has the potential to be applied to a wide range of tasks and applications. The flexibility and adaptability of the proposed TL (S2) method allow exploration and investigation of various domains, as validated through the significant improvements observed in the CT case.

The next step is to include most grayscale medical modalities (MRI, CT, and ultrasound) in the source of TL to cover most grayscale medical applications. Thus, this type of TL will offer a better generalisation of features and will be able to be used for any grayscale medical modalities.

## Figures and Tables

**Figure 12:**
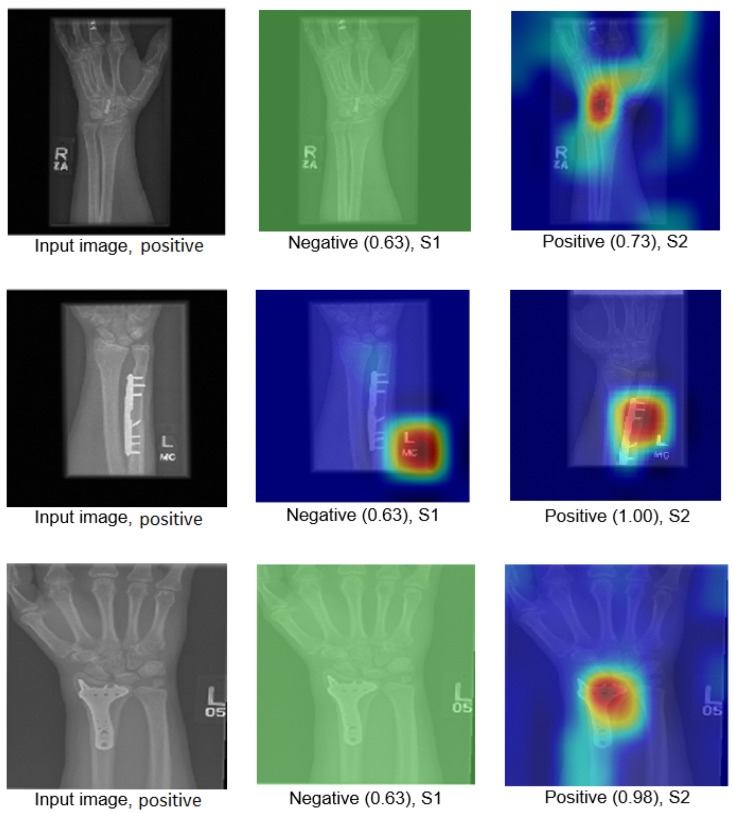
Grad-CAM and score Grad-CAM for the wrist X-ray images. The correct classification is positive.

**Figure 13:**
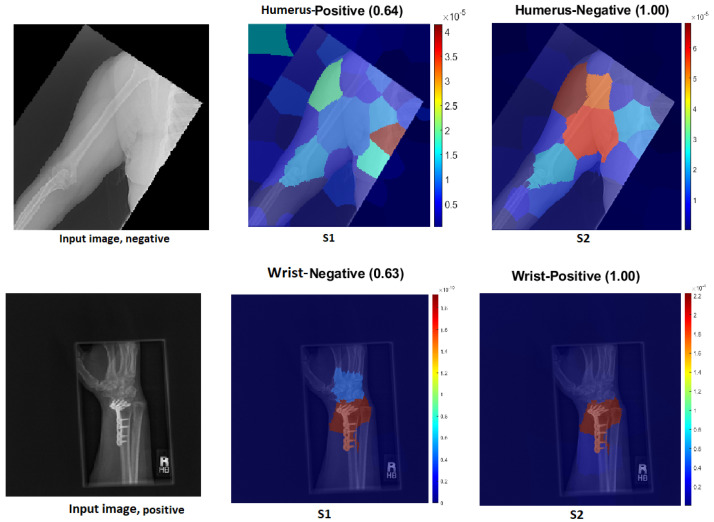
LIME and score LIME for the humerus and wrist X-ray images. The correct classification is negative for the humerus and positive for the wrist.

**Table 1:** Number of images in the MURA dataset.

Class	Training		Testing	
-	Negative	Positive	Negative	Positive
Elbow	2925	2006	234	230
Finger	3138	1968	214	247
Hand	4059	1484	271	189
Humerus	673	599	148	140
Forearm	1164	661	150	151
Shoulder	4211	4168	285	278
Wrist	5765	3987	364	295

**Table 2:** Selected models of pre-trained deep neural networks.

Model	Input Size	Parameters $10^{6}$	Depth
InceptionResNetV2	299 × 299 × 3	55.9	164
Xception	299 × 299 × 3	22.9	71

**Table 3:** The results of the DL models on the test set of the MURA dataset for the humerus task on Xception and InceptionResNetV2.

Evaluation Metric (%)	Xception	
**-**	**S1**	**S2**
**Accuracy**	69.10	84.72
**Recall**	64.29	89.29
**Precision**	69.77	81.17
**Specificity**	73.65	80.41
**F1-score**	66.91	85.03
**Cohen’s Kappa**	38.02	69.83
	InceptionResNetV2	
**Accuracy**	80.21	86.11
**Recall**	74.29	85.00
**Precision**	83.81	86.23
**Specificity**	85.81	87.16
**F1-score**	78.49	85.61
**Cohen’s Kappa**	60.27	72.20

**Table 4:** The results of the DL models on the test set of the MURA dataset for the wrist task on Xception and InceptionResNetV2.

Evaluation Metric (%)	Xception	
**-**	**S1**	**S2**
**Accuracy**	69.10	84.07
**Recall**	64.29	73.56
**Precision**	69.77	88.93
**Specificity**	73.65	92.58
**F1-score**	66.91	80.52
**Cohen’s Kappa**	38.02	68.11
	InceptionResNetV2	
**Accuracy**	80.21	82.85
**Recall**	74.29	67.80
**Precision**	83.81	91.74
**Specificity**	85.81	95.05
**F1-score**	78.49	77.97
**Cohen’s Kappa**	60.27	64.45

**Table 5:** The results of feature fusion with the coarse KNN classifier on a test set of the MURA dataset for the humerus task.

Evaluation Metric (%)	Two Models and the Coarse KNN Classifier	
***-***	**S1**	**S2**
**Accuracy**	84.03	87.85
**Recall**	80.71	88.57
**Precision**	85.61	86.71
**Specificity**	87.16	87.16
**F1-score**	83.09	87.63
**Cohen’s Kappa**	67.98	75.69

**Table 6:** The results of feature fusion with the Gaussian naive Bayes classifier on a test set of the MURA dataset for the humerus task.

Evaluation Metric (%)	Two Models and the Gaussian Naive Bayes Classifier	
***-***	**S1**	**S2**
**Accuracy**	80.60	86.80
**Recall**	80.50	86.90
**Precision**	80.60	86.90
**Specificity**	80.50	86.90
**F1-score**	80.50	86.90
**Cohen’s Kappa**	61.05	73.64

**Table 7:** The results of feature fusion with the coarse KNN Classifier on a test set of the MURA dataset for the wrist task.

Evaluation Metric (%)	Two Models and the Coarse KNN Classifier	
***-***	**S1**	**S2**
**Accuracy**	81.64	85.58
**Recall**	76.27	76.95
**Precision**	81.52	89.37
**Specificity**	85.99	92.58
**F1-score**	78.81	82.70
**Cohen’s Kappa**	62.64	70.46

**Table 8:** The results of feature fusion with the linearSVM classifier on a test set of the MURA dataset for the wrist task.

Evaluation Metric (%)	Two Models and the LinearSVM Classifier	
***-***	**S1**	**S2**
**Accuracy**	81.20	85.00
**Recall**	80.40	84.20
**Precision**	80.40	85.50
**Specificity**	80.40	84.20
**F1-score**	81.00	84.90
**Cohen’s Kappa**	61.48	69.26

**Table 9:** Comparison against the state-of-the-art methods considering the test set of the MURA dataset for the humerus detection task.

Authors	Accuracy	Recall	Precision	Specificity	F1-Score	Cohen’s Kappa
**Ibrahem et al.** [59]	82.08%	81.01%	80.60%	83.21%	80.80%	64.17%
**Huynh et al.** [60]	68.40%	64.00%	68.70%	72.20%	70.40%	-
**Olczak et al.** [61]	83.00%	-	-	-	-	-
**Luong et al.** [62]	84.00%	-	-	-	-	-
**This Study**	87.85%	88.57%	86.71%	87.16%	87.63%	75.69%

**Table 10:** Comparison against the state-of-the-art methods considering the test set of the MURA dataset for the wrist detection task.

Authors	Accuracy	Recall	Precision	Specificity	F1 Score	Cohen’s Kappa
**Ibrahem et al.** [59]	82.79%	89.89%	87.38%	71.80%	88.60%	64.60%
**Mall et al.** [63]	62.00%	35.40%	60.50%	82.30%	44.70%	-
**Karam et al.** [64]	74.91%	61.98%	72.11%	-	66.66%	-
**Saadawy et al.** [65]	73.42%	-	-	-	-	-
**Nazim et al.** [66]	78.10%	-	-	-	-	-
**Dang et al.** [67]	79.00%	-	-	-	-	
**This Study**	85.58%	76.95%	89.37%	92.58%	82.70%	70.46%

**Table 11:** The results of the DL models on the test dataset of chest CT scan on Xception and InceptionResNetV2.

Evaluation Metric (%)	Xception	
***-***	**S1**	**S2**
**Accuracy**	96.65	99.30
**Recall**	95.30	99.40
**Precision**	97.40	99.20
**Specificity**	95.30	99.40
**F1-score**	96.30	99.30
**Cohen’s Kappa**	92.34	98.57
**Evaluation Metric (%)**	**InceptionResNetV2**	
**-**	**S1**	**S2**
**Accuracy**	96.65	98.50
**Recall**	95.30	98.20
**Precision**	97.40	98.60
**Specificity**	95.30	98.20
**F1-score**	96.30	98.40
**Cohen’s Kappa**	92.34	96.77

## Data Availability

https://stanfordmlgroup.github.io/competitions/mura/ (accessed on 23 March 2023).
